# 2016 Audited schedule of changes in net assets

**DOI:** 10.5195/jmla.2017.340

**Published:** 2017-10-01

**Authors:** Ray Naegele

The table below summarizes the association’s financial status as of December 31, 2016. For a more complete audit report and related information, see the 2015/16 MLA Annual Report (members only). This report includes balance sheets, fund status reports, budgeted and actual revenues and expenditures, and a schedule of investments. Members may obtain a copy of the audit report from MLA headquarters.

**Table 1:**
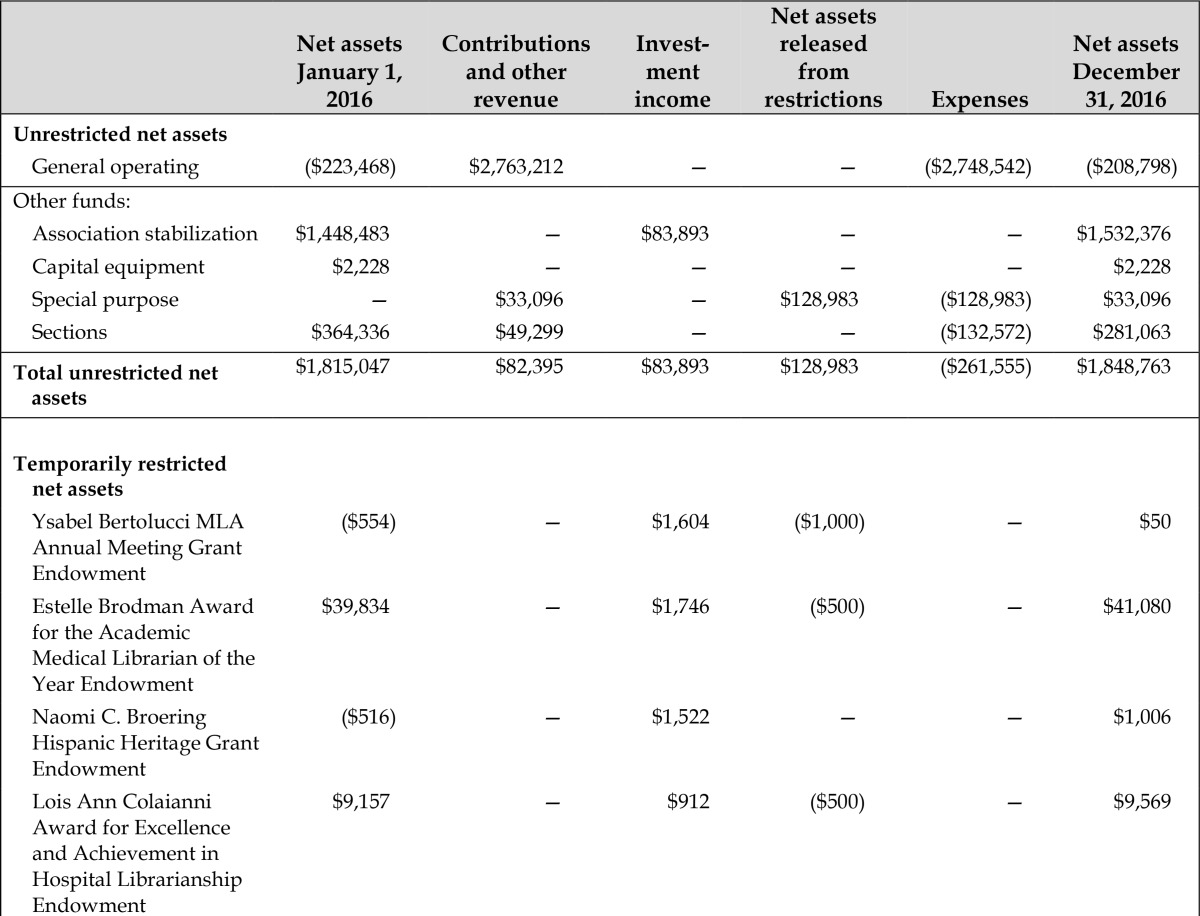

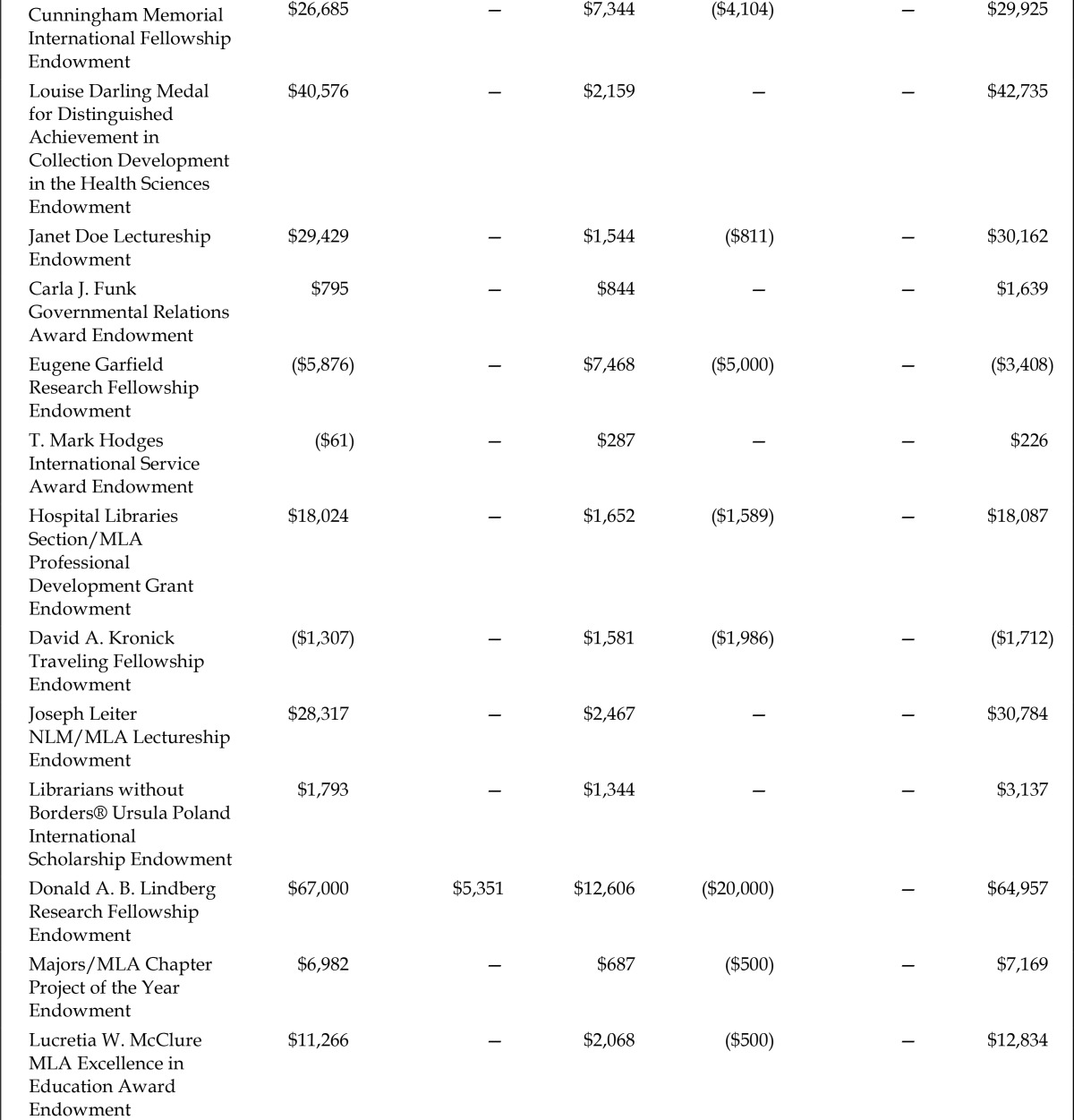

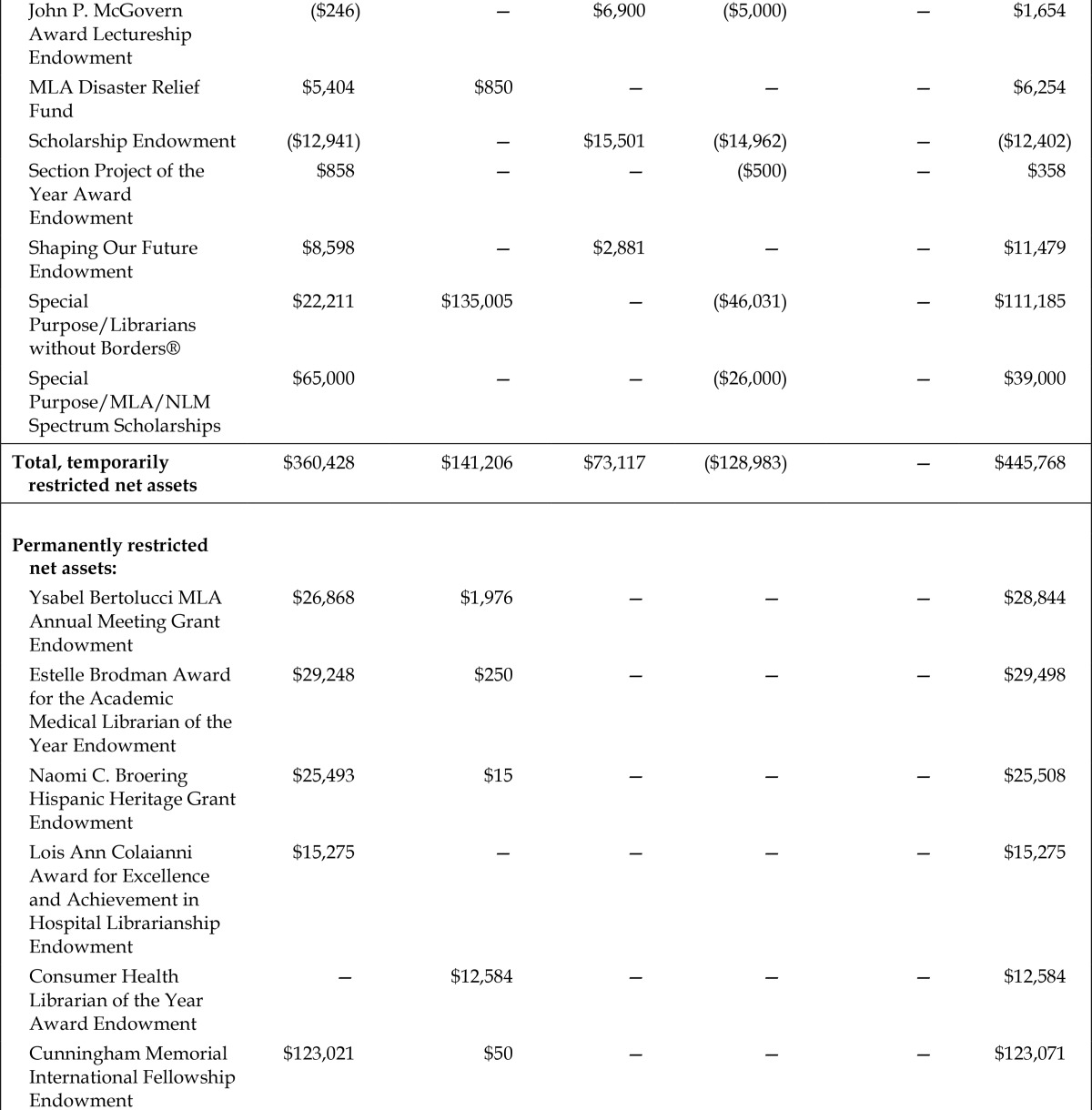

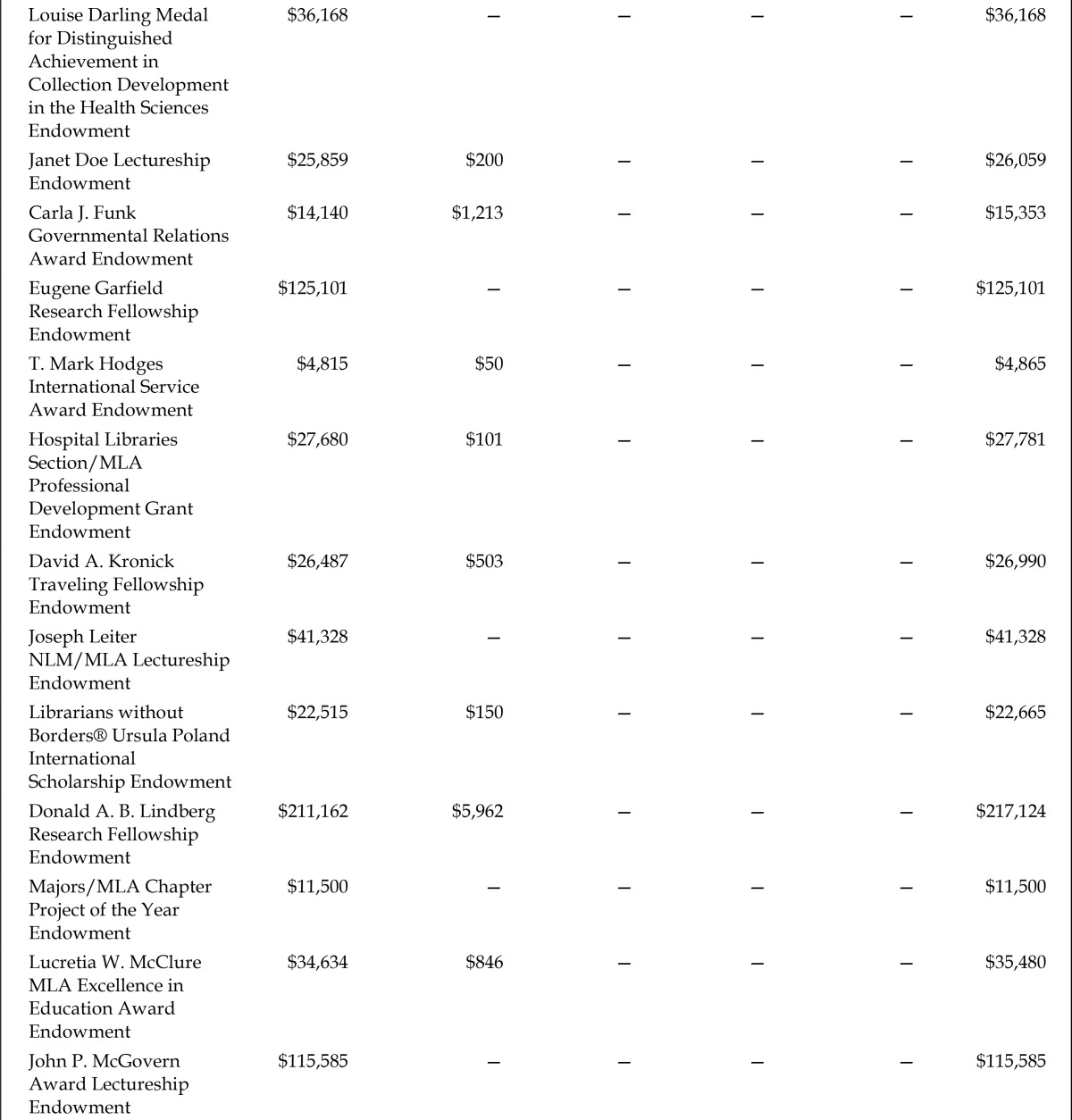
Medical Library Association schedule of changes in net assets by fund year ended December 31, 2016

	Net assets January 1, 2016	Contributions and other revenue	Investment income	Net assets released from restrictions	Expenses	Net assets December 31, 2016
**Unrestricted net assets**						
General operating	($223,468)	$2,763,212	—	—	($2,748,542)	($208,798)

Other funds:						
Association stabilization	$1,448,483	—	$83,893	—	—	$1,532,376
Capital equipment	$2,228	—	—	—	—	$2,228
Special purpose	—	$33,096	—	$128,983	($128,983)	$33,096
Sections	$364,336	$49,299	—	—	($132,572)	$281,063

**Total unrestricted net assets**	$1,815,047	$82,395	$83,893	$128,983	($261,555)	$1,848,763

**Temporarily restricted net assets**						
Ysabel Bertolucci MLA Annual Meeting Grant Endowment	($554)	—	$1,604	($1,000)	—	$50
Estelle Brodman Award for the Academic Medical Librarian of the Year Endowment	$39,834	—	$1,746	($500)	—	$41,080
Naomi C. Broering Hispanic Heritage Grant Endowment	($516)	—	$1,522	—	—	$1,006
Lois Ann Colaianni Award for Excellence and Achievement in Hospital Librarianship Endowment	$9,157	—	$912	($500)	—	$9,569
Cunningham Memorial International Fellowship Endowment	$26,685	—	$7,344	($4,104)	—	$29,925
Louise Darling Medal for Distinguished Achievement in Collection Development in the Health Sciences Endowment	$40,576	—	$2,159	—	—	$42,735
Janet Doe Lectureship Endowment	$29,429	—	$1,544	($811)	—	$30,162
Carla J. Funk Governmental Relations Award Endowment	$795	—	$844	—	—	$1,639
Eugene Garfield Research Fellowship Endowment	($5,876)	—	$7,468	($5,000)	—	($3,408)
T. Mark Hodges International Service Award Endowment	($61)	—	$287	—	—	$226
Hospital Libraries Section/MLA Professional Development Grant Endowment	$18,024	—	$1,652	($1,589)	—	$18,087
David A. Kronick Traveling Fellowship Endowment	($1,307)	—	$1,581	($1,986)	—	($1,712)
Joseph Leiter NLM/MLA Lectureship Endowment	$28,317	—	$2,467	—	—	$30,784
Librarians without Borders® Ursula Poland International Scholarship Endowment	$1,793	—	$1,344	—	—	$3,137
Donald A. B. Lindberg Research Fellowship Endowment	$67,000	$5,351	$12,606	($20,000)	—	$64,957
Majors/MLA Chapter Project of the Year Endowment	$6,982	—	$687	($500)	—	$7,169
Lucretia W. McClure MLA Excellence in Education Award Endowment	$11,266	—	$2,068	($500)	—	$12,834
John P. McGovern Award Lectureship Endowment	($246)	—	$6,900	($5,000)	—	$1,654
MLA Disaster Relief Fund	$5,404	$850	—	—	—	$6,254
Scholarship Endowment	($12,941)	—	$15,501	($14,962)	—	($12,402)
Section Project of the Year Award Endowment	$858	—	—	($500)	—	$358
Shaping Our Future Endowment	$8,598	—	$2,881	—	—	$11,479
Special Purpose/Librarians without Borders®	$22,211	$135,005	—	($46,031)	—	$111,185
Special Purpose/MLA/NLM Spectrum Scholarships	$65,000	—	—	($26,000)	—	$39,000

**Total, temporarily restricted net assets**	$360,428	$141,206	$73,117	($128,983)	—	$445,768

**Permanently restricted net assets:**						
Ysabel Bertolucci MLA Annual Meeting Grant Endowment	$26,868	$1,976	—	—	—	$28,844
Estelle Brodman Award for the Academic Medical Librarian of the Year Endowment	$29,248	$250	—	—	—	$29,498
Naomi C. Broering Hispanic Heritage Grant Endowment	$25,493	$15	—	—	—	$25,508
Lois Ann Colaianni Award for Excellence and Achievement in Hospital Librarianship Endowment	$15,275	—	—	—	—	$15,275
Consumer Health Librarian of the Year Award Endowment	—	$12,584	—	—	—	$12,584
Cunningham Memorial International Fellowship Endowment	$123,021	$50	—	—	—	$123,071
Louise Darling Medal for Distinguished Achievement in Collection Development in the Health Sciences Endowment	$36,168	—	—	—	—	$36,168
Janet Doe Lectureship Endowment	$25,859	$200	—	—	—	$26,059
Carla J. Funk Governmental Relations Award Endowment	$14,140	$1,213	—	—	—	$15,353
Eugene Garfield Research Fellowship Endowment	$125,101	—	—	—	—	$125,101
T. Mark Hodges International Service Award Endowment	$4,815	$50	—	—	—	$4,865
Hospital Libraries Section/MLA Professional Development Grant Endowment	$27,680	$101	—	—	—	$27,781
David A. Kronick Traveling Fellowship Endowment	$26,487	$503	—	—	—	$26,990
Joseph Leiter NLM/MLA Lectureship Endowment	$41,328	—	—	—	—	$41,328
Librarians without Borders® Ursula Poland International Scholarship Endowment	$22,515	$150	—	—	—	$22,665
Donald A. B. Lindberg Research Fellowship Endowment	$211,162	$5,962	—	—	—	$217,124
Majors/MLA Chapter Project of the Year Endowment	$11,500	—	—	—	—	$11,500
Lucretia W. McClure MLA Excellence in Education Award Endowment	$34,634	$846	—	—	—	$35,480
John P. McGovern Award Lectureship Endowment	$115,585	—	—	—	—	$115,585
Scholarship Endowment	$259,667	$4,287	—	—	—	$263,954
Shaping Our Future Endowment	$48,269	$2,335	—	—	—	$50,604

**Total, permanently restricted net assets**	$1,224,815	$30,522	—	—	—	$1,255,337

**Total all net assets**	**$3,176,822**	**$3,017,335**	**$157,010**	**$-**	**($3,010,097)**	**$3,341,070**

Additional information regarding MLA finances appears in the May 2017 issue of the *MLA News*.

